# Risk factors for idiopathic central precocious puberty of girls in Fujian province

**DOI:** 10.1186/1687-9856-2015-S1-P94

**Published:** 2015-04-28

**Authors:** Ruimin Chen, Chunyan Cai, Zhijian Hu, Xianquan Lin, Yunfei Li, Ying Zhang, Xiaohong Yang, Xin Yuan

**Affiliations:** 1 Fuzhou Children’s Hospital of Fujian, Fuzhou, Fujian, China; 2 Fujian Medical University, Fuzhou, Fujian, China

**Keywords:** precocious puberty, risk factors, Logistic Regression analysis

## Objective

This study aimed to investigate the potential risk factors of idiopathic central precocious puberty (ICPP) girls in Fujian province.

## Methods

A case-control study was conducted in 566 girls who were diagnosed with ICPP at endocrinology department and 547 healthy girls for routine physical examination in Fuzhou Children’s Hospital of Fujian from October 2011 to October 2013. Parents were asked to fulfill the questionnaires on children’s diets, behaviors, parents and family conditions, and Logistic regression analysis was conducted for risk factors.

## Results

Chi-square test showed that 23 variables had significant statistically difference. Logistic regression analysis indicated that some variables were entered the final model: mother's age at menarche were older than 13 years (OR=0.278, 95%CI: 0.201,0.384), intaking organic fruits (OR=0.316, 95%CI: 0.157,0.634), daily exercise time(OR=0.609, 95%CI: 0.490,0.758), intaking ordinary vegetables (OR=1.275, 95%CI: 1.095,1.485), intaking general livestock (OR=1.364, 95%CI: 1.199,1.551), BMI(OR=1.599, 95%CI: 1.365,1.874), income of parents (OR=1.671, 95%CI: 1.317,2.120), heavy study burden (OR=1.818, 95%CI: 1.121,2.948), intaking instant food(OR=2.990, 95%CI: 1.241,7.203), intaking nourishment (OR=3.736, 95%CI: 2.063,6.765), using adult cosmetics (OR=5.284, 95%CI: 3.240,8.618). Among the variables, mother's age at menarche were older than 13 years, intaking organic fruit and doing more exercise for a long time were demonstrated to be the protective factors for ICPP, and others were risk factors.

## Conclusion

There were many factors related to ICPP girls in Fujian province, such as mother’s age at menarche, diet and behaviors, BMI, parental income, and heavy study burden.

